# A Study of Dyslipidemia and Its Clinical Implications in Childhood Nephrotic Syndrome

**DOI:** 10.7759/cureus.47434

**Published:** 2023-10-21

**Authors:** Pritikar Dowerah, Arpita Gogoi, Caroline D Shira, Bikash Sarkar, Sarada Mazumdar

**Affiliations:** 1 Pediatrics and Neonatology, Assam Medical College & Hospital, Dibrugarh, IND

**Keywords:** ldl cholesterol, hdl cholesterol, kidney disease, nephrotic-range proteinuria, idiopathic nephrotic syndrome, awareness of cardiovascular disease, pediatric hyperlipidemia, pattern of dyslipidemia, steroid resistant nephrotic syndrome, childhood nephrotic syndrome

## Abstract

Background: Nephrotic syndrome in children is characterized by dyslipidemia, which is an indirect risk factor for cardiovascular diseases, progressive glomerulosclerosis, and related complications. The objective is to determine the range of lipid profile abnormalities in relation to onset, relapse, and remission, as well as to determine if there is any relationship between dyslipidemia and the frequency of relapses.

Methods: One hundred and two diagnosed cases of nephrotic syndrome in the age group of less than 12 years were included, out of which 64 patients belonged to the first episode of nephrotic syndrome and 38 patients were relapse cases. Steroid-resistant nephrotic syndrome cases or patients with a history of diabetes mellitus, hypothyroidism, familial hypercholesterolemia, children with chronic kidney disease, and edema due to other causes were excluded from the study.

Results: There was a statistically significant increase in lipid parameters except for high-density lipoprotein (HDL) cholesterol at the disease onset when compared to remission in cases of the first episode as well as relapse cases of nephrotic syndrome. There was a positive correlation between relapse frequency and dyslipidemia. Dyslipidaemia was also associated with low serum albumin, with a p-value <0.001, which is statistically significant.

Conclusion: Dyslipidemia is significantly higher in relapse cases of nephrotic syndrome and remains higher even during remission. Dyslipidemia is a directly associated risk factor for atherosclerosis and coronary heart disease (CAD), along with progressive glomerulosclerosis. Early identification and treatment of hyperlipidemia is therefore justified so that along with longevity, we can also improve the quality of life of children suffering from nephrotic syndrome.

## Introduction

Nephrotic syndrome is a glomerular basement membrane disease that is characterized by heavy proteinuria, hypoalbuminemia (<3g/dl), edema, and hyperlipidemia (cholesterol >250mg/dl) [1].

Hyperlipidemia is caused by stimulation of lipoprotein synthesis and diminished catabolism as a result of decreased hepatic lipoprotein lipase due to hypoalbuminemia and increased intrahepatic expression of proprotein convertase subtilisin/kexin type 9 (PCSK9), which results in degradation of low-density lipoprotein receptor (LDLR), thereby causing impaired low-density lipoprotein (LDL) clearance. Hypoalbuminemia produces an imbalance in the ratio of free fatty acids to proteins in the blood, which stimulates angiopoietin-like four levels to rise and downregulates hepatic lipase, resulting in poor intermediate-density lipoprotein (IDL) clearance [2,3,4]. Therefore, dyslipidemia in nephrotic syndrome raises total cholesterol, triglycerides, LDL, very low-density lipoprotein (VLDL), and IDL, but high-density lipoprotein (HDL) is either normal, low, or raised. [5,6,7]. Dyslipidemia in cases of minimal change nephrotic syndrome (MCNS) is seen during the active phase of the disease [8]. According to one study, dyslipidemia in a non-diabetic adult with nephrotic syndrome is associated with an increased risk for myocardial infarction (MI) by 5.5 times, with a confidence interval (CI) of 95%, as compared to adults without nephrotic syndrome [9]. With an increase in awareness, early diagnosis, and management of nephrotic syndrome, most patients survive the adolescent period. However, death due to complications of nephrotic syndrome, such as cardiovascular disease (CVD), becomes a concern as hyperlipidemia has been found to be indirectly associated with CVD. [10]. Several experiments have shown that dyslipidemia, along with proteinuria, leads to glomerulosclerosis by directly affecting the basement membrane, which is termed the “nephrotoxicity hypothesis” [2,11].

Hence, the main objective of our study is to determine the range of lipid profile abnormalities in nephrotic syndrome depending on the stages of the disease, such as onset, relapse, and remission, as well as to determine whether there is, if any, a relationship between the severity of dyslipidemia and the frequency of relapses in nephrotic syndrome.

## Materials and methods

This hospital-based observational study was conducted in the pediatrics department of Assam Medical College and Hospital, Dibrugarh, Assam, India, over a period of one year, from June 2018 until May 31, 2019. The study was started after receiving due approval from the ethics committee of Assam Medical College (approval no. AMC/EC/PG/2602, dated April 18, 2018). One hundred and two diagnosed cases of nephrotic syndrome (both initial episodes and relapse cases), aged less than 12 years, were enrolled after taking written informed consent. Children with steroid-resistant nephrotic syndrome, chronic kidney disease, and a history of diabetes mellitus, hypothyroidism, or familial hypercholesterolemia were excluded. Children with edema due to other causes such as congestive cardiac failure, protein-energy malnutrition, and chronic liver disease were also excluded from the study. Table 1 shows the case definitions that were used in this study.

**Table 1:** Case definitions used in the study

	Details
Initial episode	Child presenting with nephrotic range proteinuria (3.5 g/24 hours) or a spot urine protein: creatinine ratio >2, hypoalbuminemia (<2.5 g/dl), edema, hypercholesterolemia (>200mg/dl)
Remission	Urine albumin nil or trace (or proteinuria <4 mg/m^2^/hr) in an early morning urinary sample for three consecutive days.
Relapse	Urinary albumin 3+/4+ (or proteinuria 40 mg/m^2^/hr) in an early morning urinary sample for three consecutive days for a patient who has been in remission previously.
Frequent relapse	Two or more relapses in the initial six months or four or more relapses in any 12 months.
Steroid dependence	Two consecutive relapses when on alternate-day steroids or within 14 days of its discontinuation.
Steroid resistance	Absence of remission despite daily steroid therapy at 2 mg/kg/day for four weeks and alternate days for the next four weeks.

Sample collection

After eight hours of overnight fasting, approximately 2 ml of blood was collected from children with the first episode and relapse cases of nephrotic syndrome, first at the time of admission and again at remission, in sterile empty vials (SEV) and was then allowed to clot. The clotted sample was then centrifuged at 3000 rps for about three to five minutes, and then the supernatant serum was taken to perform the various tests. The estimation of total cholesterol and serum triglyceride was done enzymatically via a series of coupled reactions followed by their absorbance at 500 nm. The color intensity was found to be proportional to the concentration. Blocking agents such as alpha (α)-cyclodextrin were used when measuring serum HDL. Alpha-cyclodextrin forms complexes with apo B-containing lipoproteins in the presence of Mg+2, rendering them unreactive, thereby effectively excluding HDL cholesterol and detecting it only enzymatically. Since circulating cholesterol is found in three major lipoproteins, namely VLDL, LDL, and HDL, the following relationships were used:

Total cholesterol = [VLDL cholesterol] + [LDL cholesterol] + [HDL cholesterol]

LDL cholesterol = [Total cholesterol] - [HDL cholesterol] - [Triglyceride] / 5,

where triglyceride 5 is an estimate of VLDL cholesterol and all values are expressed in mg/dl.

MicroLab 300 S.No. 14-5768, available at the Department of Biochemistry, Assam Medical College, Dibrugarh, was used for lipid estimation. IBM Statistical Package of Social Sciences software for Windows, version 21.0 (IBM Corp., Armonk, NY), and Microsoft Excel 2010 (Microsoft Corp., Redmond, WA) were used for the statistical analysis of the data. The statistical significance was expressed in terms of a p-value fixed at 5% (p-value <0.05). Continuous data results were presented as mean +/- standard deviation and compared using ANOVA. Association among continuous variables was measured using Pearson’s correlation coefficient (r).

## Results

A total of 102 cases of nephrotic syndrome under 12 years of age were enrolled in the study, of which n=28 (27.45%) were in the age group of 0 to four years, n=56 (54.9%) in the age group of five to eight years, and n=18 (17.65%) in the age group of eight to 12 years (Table 2).

**Table 2:** Age-wise distribution of patients

Age group (in years)	Number (n)	Percentage (%)	Mean ± S.D.
0–4	28	27.45	6.20 ± 2.21 years
5–8	56	54.90	
9–12	18	17.65	
Total	102	100.00	--

The mean age of presentation was 6.20 ± 2.21 years. Hence, the majority of nephrotic patients visiting the department of pediatrics at Assam Medical College and Hospital belonged to the school-going age group. Based on gender, male children outnumbered female children, with n=59 (57.84%) males against n=43 (42.16%) females. Idiopathic nephrotic syndrome was found to be more prevalent in boys than in girls (1.3:1) in this study (Table 3).

**Table 3:** Sex-wise distribution of patients

Sex	Number (n)	Percentage (%)	Ratio (Male : Female)
Male	59	57.84	1.37:1
Female	43	42.16	
Total	102	100.00	

Out of 102 patients, n=64 (62.75%) patients belonged to the first episode of nephrotic syndrome, and n=38 (37.25%) patients were relapse cases, of which n=10 (9.8%) patients were frequent relapse cases and n=28 (27.45%) patients were non-frequent relapse cases (Table 4).

**Table 4:** Distribution of cases according to first episode and relapse

Cases	Number (n)	Percentage (%)
First episode	64	62.75
Relapse	38	
Frequent relapse	10	9.80
Non-frequent relapse	28	27.45
Total	102	100.00

In Tables 5-6, the mean total serum cholesterol, LDL cholesterol, VLDL cholesterol, HDL cholesterol, and triglyceride values have been compared according to different age groups and sexes, during the onset of disease and during remission.

**Table 5:** Comparison of lipid parameters according to age distribution at onset and at remission ANOVA test; the p-value <0.05 is considered significant LDL: low-density lipoprotein; VLDL: very low-density lipoprotein; HDL: high-density lipoprotein

	Age group (years)	Onset of the disease			At remission		
		Mean	± S.D.	p-value*	Mean	± S.D.	p-value*
Total cholesterol (mg/dl)	0–4	398.68	101.02	0.099	294.43	89	0.077
	5–8	441.75	113.49		337.52	93.8	
	9–12	463.67	93.96		347.22	81.81	
LDL cholesterol (mg/dl)	0–5	291.33	91.5	0.100	202.31	82.95	0.098
	5–9	330.71	103.19		239.15	83.74	
	9–13	350.06	85.13		248.12	74.76	
VLDL cholesterol (mg/dl)	0–6	57.17	19.44	0.096	37.54	13.76	0.054
	5–10	62.4	19.61		43.85	16.23	
	9–14	69.94	18.1		48.43	14.27	
HDL cholesterol (mg/dl)	0–7	50.18	10.98	0.103	54.57	8.88	0.165
	5–11	48.64	9.53		54.52	7.12	
	9–15	43.67	11.32		50.67	7.87	
Triglyceride (mg/dl)	0–8	285.86	97.19	0.096	187.71	68.79	0.054
	5–12	312	98.06		219.23	81.13	
	9–16	349.72	90.48		242.17	71.33	

**Table 6:** Comparison of lipid parameters at onset and at remission (according to sex) The Student t-test is significant at p <0.05. LDL: low-density lipoprotein; VLDL: very low-density lipoprotein; HDL: high-density lipoprotein

			Onset of the disease	p-value	At remission	p-value
Total cholesterol (mg%)	Male	Mean	425.76	0.306101	321.06	0.3517
		SD	107.96		89.34	
	Female	Mean	449.17		339.54	
		SD	109.32		97.04	
LDL cholesterol (mg%)	Male	Mean	316.40	0.326036	225.55	0.40829
		SD	99.35		81.22	
	Female	Mean	336.55		240.33	
		SD	96.82		87.12	
VLDL cholesterol (mg%)	Male	Mean	60.99	0.372605	41.51	0.21772
		SD	18.79		15.07	
	Female	Mean	64.79		45.64	
		SD	21.05		16.35	
HDL cholesterol (mg%)	Male	Mean	48.37	0.808507	54.00	0.79822
		SD	10.13		7.72	
	Female	Mean	47.83		53.57	
		SD	11.03		8.15	
Triglycerides (mg%)	Male	Mean	304.96	0.372605	207.54	0.21772
		SD	93.96		75.33	
	Female	Mean	323.97		228.20	
		SD	105.27		81.23	

The differences in cholesterol, LDL cholesterol, VLDL cholesterol, HDL cholesterol, and triglyceride levels were found to be statistically insignificant at the onset of the disease and at remission, respectively. Also, no statistically significant difference was noted in cholesterol, LDL cholesterol, VLDL cholesterol, HDL cholesterol, or triglyceride levels in male and female patients before and after treatment.

Table 7 shows the comparison of lipid profiles at the onset of the disease and at remission in patients with the first episode and relapse cases of nephrotic syndrome.

**Table 7:** Comparison of lipid parameters in first-episode and relapse cases of nephrotic syndrome LDL: low-density lipoprotein; VLDL: very low-density lipoprotein; HDL: high-density lipoprotein

	Total cholesterol (MG%)		LDL cholesterol (MG%)		VLDL cholesterol (MG%)		HDL cholesterol (MG%)		Triglyceride (MG%)	
First episode	Onset of the disease	At remission	Onset of the disease	At remission	Onset of the disease	At remission	Onset of the disease	At remission	Onset of the disease	At remission
	383.39	72.41	279.79	68.16	55.67	15.77	49.84	9.9	278.33	78.85
	285.08	58.44	195.52	56.83	36.12	9.56	52.44	7.53	180.59	47.78
p-value	<0.001		<0.001		<0.001		0.0970		<0.001	
Relapse	518.68	106.72	396.62	98.83	73.46	20.46	48.61	11.32	367.32	102.31
	398.68	94.72	289.74	87.51	54.39	17.04	52.24	8.37	271.95	85.21
p-value	<0.001		<0.001		<0.001		0.1375		<0.001	

In the first episode of nephrotic syndrome, the difference was found to be statistically significant for all the parameters of the lipid profile at the onset and after remission, with a p-value <0.001 except for HDL cholesterol. In first-episode patients, HDL cholesterol at the onset of the disease was 49.84 ±9.90 and in remission was 52.44±7.53. The p-value for HDL cholesterol between the two groups was 0.0970, which is statistically insignificant. In relapse cases of nephrotic syndrome also, statistically significant differences were seen for total cholesterol, LDL cholesterol, VLDL cholesterol, and triglyceride levels at the onset and after remission of the disease, with a p-value <0.001 for all the parameters of the lipid profile. In the relapse group, HDL cholesterol at the onset of the disease was 48.61±11.32, and in remission was 52.24±8.37. The difference was not significant between the two groups.

Table 8 shows the comparison of lipid profiles at the onset of the disease and at remission in steroid-responsive cases and steroid-dependent cases.

**Table 8:** Comparison of lipid profiles in steroid-responsive and steroid-dependent cases of nephrotic syndrome *Student's t-test; the p-value is significant at a 5% level of significance. LDL: low-density lipoprotein; VLDL: very low-density lipoprotein; HDL: high-density lipoprotein

Lipid profile		Onset of the disease		At remission		p-value
		Mean	± S.D.	Mean	± S.D.	
Steroid-responsive	Total cholesterol (mg%)	425.86	105.35	320.73	89.54	<0.001
	LDL cholesterol (mg%)	316.53	96.04	224.84	81.32	<0.001
	VLDL cholesterol (mg%)	60.78	18.99	41.72	15.02	<0.001
	HDL cholesterol (mg%)	48.55	10.41	50.8	7.82	0.054
	Triglycerides (mg%)	303.91	94.95	208.58	75.08	<0.001
Steroid-dependent	Total cholesterol (mg%)	560.67	78.97	434.17	64.1	0.003
	LDL cholesterol (mg%)	431.8	75.14	323.23	55.76	0.006
	VLDL cholesterol (mg%)	86.53	11.91	62.27	11.44	0.001
	HDL cholesterol (mg%)	46.33	8.98	48.67	6.53	0.561
	Triglycerides (mg%)	432.67	59.57	311.33	57.18	0.001

In steroid-responsive cases, except for HDL cholesterol, the difference was found to be statistically significant for all the parameters of the lipid profile in the two groups, with a p-value <0.001. In steroid-responsive cases, HDL cholesterol at the onset of the disease was 48.55±10.41, and in remission was 50.8±7.82. The p-value for HDL cholesterol between the two groups was 0.0535, which is statistically insignificant. In steroid-dependent cases, the difference in total cholesterol, LDL cholesterol, VLDL cholesterol, and triglyceride was found to be statistically significant in the two groups with a p-value <0.001. However, the difference in HDL cholesterol level at the onset of disease and at remission was statistically insignificant with a p-value of 0.5606.

Table 9 shows a comparison of lipid profiles in frequent relapse and infrequent relapse cases, at the onset of disease and during remission.

**Table 9:** Comparison of lipid profiles in frequent and infrequent relapse cases of nephrotic syndrome *Student's t-test; the p-value is significant at a 5% level of significance. LDL: low-density lipoprotein; VLDL: very low-density lipoprotein; HDL: high-density lipoprotein

Lipid profile		Onset of the disease		At remission		p-value
		Mean	± S.D.	Mean	± S.D.	
Frequent relapse	Total cholesterol (mg%)	615.5	79.44	495.9	61.26	0.001
	LDL cholesterol (mg%)	486.02	74.88	379.02	53.74	0.002
	VLDL cholesterol (mg%)	92.28	14.7	70.48	15.06	0.004
	HDL cholesterol (mg%)	46.2	9.53	51.4	5.72	0.156
	Triglycerides (mg%)	461.4	73.51	352.4	75.28	0.004
Infrequent relapse	Total cholesterol (mg%)	484.11	93.76	363.96	79.34	<0.001
	LDL cholesterol (mg%)	364.69	86.59	257.86	74.33	<0.001
	VLDL cholesterol (mg%)	66.74	17.99	48.64	13.87	<0.001
	HDL cholesterol (mg%)	47.90	8.94	51.90	7.19	0.071
	Triglycerides (mg%)	333.71	89.96	243.21	69.33	<0.001

In frequent relapse cases, all the lipid parameters showed a significant difference between the two groups except for HDL cholesterol. At the onset of the disease, HDL cholesterol was 46.2±9.53, and at remission, it was 51.4±5.72, and this difference was statistically insignificant in the two groups with a p-value of 0.1563. In infrequent relapse cases, the total mean serum cholesterol, LDL cholesterol, VLDL cholesterol, HDL cholesterol, and triglyceride values were found to be 484.11±93.76, 364.69±86.59, 66.74±17.99, 47.9±8.94, and 333.71±89.96, respectively, at the onset of the disease in comparison to 363.96±79.34, 257.86±74.33, 48.64±13.87, 51.9±7.19, and 243.21±69.33 at remission. The difference in all these lipid parameters was statistically significant with a p-value <0.001, except for HDL cholesterol.

A correlation between the frequency of relapses and the lipid profile was done and is depicted in Table 10.

**Table 10:** Correlation between the number of relapses and dyslipidemia Total cholesterol, LDL cholesterol, VLDL cholesterol, and triglyceride levels showed a strongly positive correlation with the number of relapses with a significant p-value of < 0.001, and HDL cholesterol showed a negative correlation with the number of relapses with an r-value of -0.8320. LDL: low-density lipoprotein; VLDL: very low-density lipoprotein; HDL: high-density lipoprotein

Lipid profile	Number of relapses	
	r-value	p-value
Total cholesterol (mg%)	0.854	< 0.001
LDL cholesterol (mg%)	0.837	< 0.001
VLDL cholesterol (mg%)	0.871	< 0.001
HDL cholesterol (mg%)	-0.832	< 0.001
Triglycerides (mg%)	0.871	< 0.001

**Figure 1:**
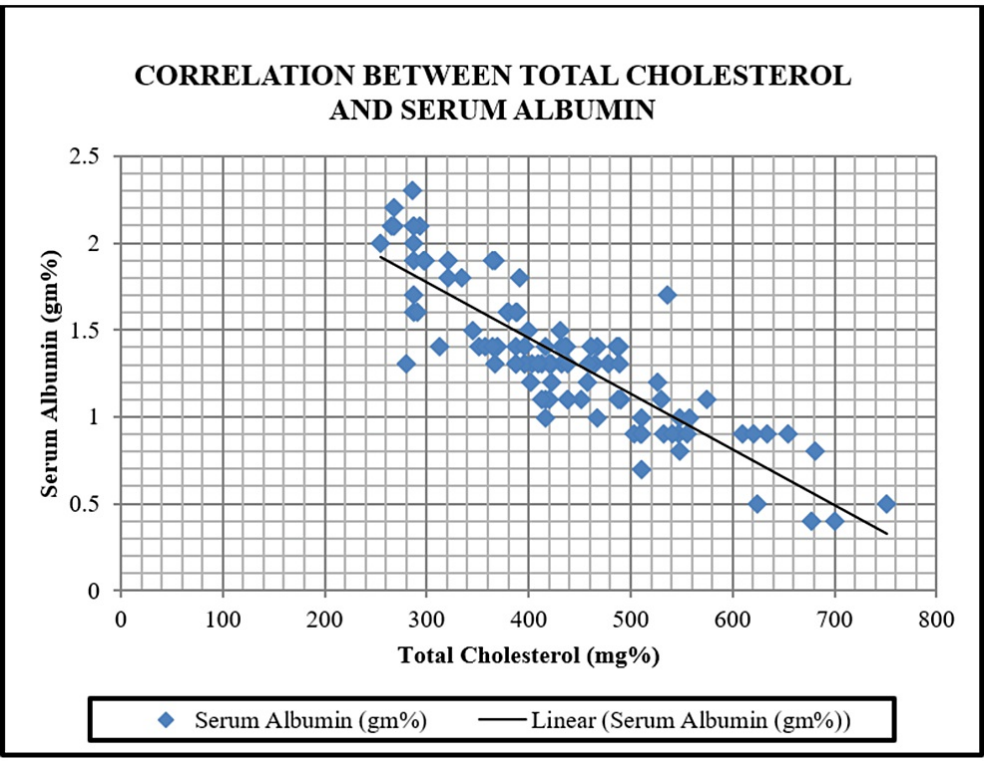
Correlation between total cholesterol and serum albumin A negative correlation was seen between serum cholesterol and serum albumin, with an r-value of -0.8571. The correlation was strongly significant, with a p-value < 0.001.

Total cholesterol, LDL cholesterol, VLDL cholesterol, and triglyceride levels showed a strongly positive correlation with the number of relapses, with a significant p-value of <0.001. So, with the increase in the number of relapses, there was an increase in all the parameters of the lipid profile. However, HDL cholesterol showed a negative correlation with the number of relapses, with an r-value of -0.8320. Another correlation between serum albumin level and serum cholesterol level was done and is represented in Figure 1.

A negative correlation was seen between serum cholesterol and serum albumin, with an r-value of -0.8571. The correlation was strongly significant, with a p-value <0.001.

## Discussion

This study suggests that dyslipidemia is found in almost all nephrotic syndrome cases, with more severity in cases of relapse as compared to the first episode. However, the lipid parameters in cases of the first episode of nephrotic syndrome decrease significantly at remission as compared to relapse cases, where the levels of lipid parameters remain higher even at remission. In relapse cases, the mean serum cholesterol value at the onset of the disease and at remission was found to be much higher than it was in first-episode cases. Similar results were found by Sreenivasa et al. in 50 steroid-sensitive nephrotic syndrome children between two and 12 years old who were evaluated for lipid derangement [7]. In the study by Dnyanesh et al., where they measured lipid levels at disease onset and remission in 30 children with nephrotic syndrome, they found significant differences in lipid parameters, except for HDL cholesterol [12]. In this study, when lipid profiles were compared at the onset of disease and at remission in cases of frequent relapse nephrotic cases, except for HDL cholesterol, all lipid parameters showed significant differences, which is also supported by the study done by Prashanth KS et al., where 75 children with nephrotic syndrome were evaluated for dyslipidemia, which again showed statistically significant differences in all lipid parameters except for HDL cholesterol [13].

The findings of this study are similar to those of a study by Sreenivasa et al. and showed a statistically significant difference in lipid parameters except for HDL cholesterol at the onset of disease and at remission in steroid-responsive cases [7]. This study also shows significant differences in lipid profiles at disease onset and in remission, except that HDL cholesterol is not statistically significant in steroid-dependent patients. This finding is supported by the data of Prashanth KS et al., who found that out of 75 children, 35 were steroid-dependent and found no significant difference in HDL cholesterol [13].

In this study, a positive correlation was found between relapse frequency and dyslipidemia with a significant p-value <0.001 and a negative correlation between HDL cholesterol and relapse frequency with an r-value of -0.8320. Similar results were found by Mahmud S et al., who observed that children who had prolonged hypercholesterolemia had more chances of relapse, which can act as a predictor in cases of idiopathic nephrotic syndrome [14]. Lawang et al., through their study, also concluded that elevated cholesterol levels during remission were associated with a significant risk of relapse [15]. In this study, a negative correlation between serum albumin and serum cholesterol (r = -0.8571) has been observed, which was statistically significant with a p-value <0.001 and is also supported by Hossain et al., where the mean serum cholesterol was 240 (±07) mg/dl and the mean serum total albumin value was 1.88 (±.37) mg/dl, with a negative correlation using Pearson’s correlation test [16]. Similar results were also found by Sreenivasa et al., who showed a highly statistically significant (p-value of 0.000) inverse relationship between cholesterol and albumin [7]. An r-value of -0.850 between serum albumin and cholesterol was reported by Reddy et al. in nephrotic patients with cholesterol levels of 459.30 mg/dl and mean albumin levels of 1.81 g/dl [17].

In this study, it was found that dyslipidemia is persistent even at remission, which may predict a protracted, frequently relapsing, or steroid-resistant course in children with steroid-sensitive nephrotic syndrome. So, this study addresses the gap in predicting the severity of future relapses based on persistent dyslipidemia at remission in both steroid-sensitive nephrotic syndrome and steroid-resistant nephrotic syndrome. Besides, this study also puts forward suggestions for carrying out further studies to find out whether lipid-lowering agents may influence reducing the frequency or steroid dependence of cases of steroid-sensitive nephrotic syndrome. This study may also enable physicians to counsel the parents of children with persistent dyslipidemia at remission about the propensity for a steroid dependence or frequently relapsing course in children with dyslipidemia and advocate more stringent follow-up and home-based urine protein monitoring to detect relapses at the early stages, before complications set in.

There are certain limitations to this study. A small sample size and a lack of long-term data might limit the generalizability of the results to a broader population. In this study, we did not follow up on the cases to evaluate the time taken for normalization of the lipid profile once the patients with nephrotic syndrome went into remission. Besides, patients with frequent relapse nephrotic syndrome were previously treated with steroid therapy, and a few of these frequent relapse cases had steroid-induced hypertension and steroid-induced obesity. Since we did not exclude patients with steroid-induced obesity and hypertension, we are unsure if hyperlipidemia occurred independently due to nephrotic syndrome, as steroid therapy in children with frequent relapse nephrotic syndrome may act as a confounder for hyperlipidemia. Longer follow-ups are needed to draw more definite conclusions.

## Conclusions

Hence, given the above results, we can say that there is significant dyslipidemia in children with nephrotic syndrome, more so in cases of relapse of nephrotic syndrome. Dyslipidemia is a directly associated risk factor for atherosclerosis and CAD, along with progressive glomerulosclerosis. We must therefore try to identify and treat hyperlipidemia early so that, along with longevity, we can also improve the quality of life. However, prospective controlled studies using lipid-lowering agents for safety and efficacy in children are required.
